# On the Expediency of an Early Delivery of the Placenta

**Published:** 1800-03

**Authors:** Thomas Peck

**Affiliations:** Higham Ferrers


### *To the Editors of the Medical and Phyſical Journal.*

GENTLEMEN,

SHOULD you think the ſubſequent obſervations deſerving a page in your Journal, you will oblige, by inſerting them,

Your's, reſpectfully,

THOMAS PECK.

*Higham Ferrers, Jan.* 16, 1800.

---

### *On the Expediency of an early Delivery of the Placenta.*

The placenta having anſwered the intention it was formed for, is found no longer ſerviceable either to the œconomy of the mother or child. It is therefore, ſeparated from the uterus, and expelled generally by the efforts of Nature, or with very little aſſiſtance. Unfortunately, however, it is ſometimes retained in utero, from the following cauſes: the rupture of the funis, or the irregular contraction of the uterus. Either of theſe cauſes exiſting, it behoves the practitioner immediately to determine his conduct: I ſay, immediately, becauſe, in my opinion, the placenta cannot be too ſpeedily removed after the expulſion of the child. I do not mean to urge the propriety of a *forcible* extraction of the placenta, the very moment the funis is divided, but to pay direct attention to the efforts of Nature; and, if ſuch efforts are not ſufficient to expel it in ten or fifteen minutes, to extract it; for, as Mr. Davies (in a former Journal*) very juſtly remarks, "hæmorrhage will not ceaſe, while the ſubſtance of the placenta is diſtending the cavity of the uterus;"— and, if it be not removed in that time, it is highly probable that the uterus will contract irregularly upon it, and occaſion ſome difficulty in its extraction. I am perſuaded, I have met with more than one inſtance in my own practice, where this has been the caſe, ariſing wholly from a delay in the extraction of the placenta. Mr. Davies ſeems aware, that manual efforts have been too forcibly and prematurely perſiſted in; I ſuppoſe he means, where there may have been an adheſion of a part of the placenta. For my own part, I never would ſuffer the ſmalleſt portion of it to *remain* in the uterus, if *manual operation* would prevent it; and, I muſt beg leave to ſay, that I conceive, if Mr. Davies had perſiſted in his attempts *at firſt*, he would have ſucceeded;

---

* Vide No. XI. page 7.

ceeded; but his patient became exhausted. I would ask him the cause of such exhaustion. Certainly, not manual efforts, but the hæmorhage; and the cause of the hæmorrhage, the retention of the placenta. It became the business, therefore, of the accoucheur, to remove the cause as speedily as *possible*, and not to regard the exhaustion of his patient *at all*, well knowing it to be *more* favourable to his attempts. Mr. D. in this exhausted state of his patient, supported her with cordials, &c. which, in the opinion of most people, must tend to increase the symptoms under which his patient laboured; I mean, the hæmorrhage and its consequences. Another objection to desisting in such case is, the probability of still greater difficulty, from the irregular contraction of the uterus. Mr. Davies will pardon the liberty I have taken, in commenting on his case: I mean, to press the necessity of an early removal of the placenta from the uterus, as, having performed its proper office, and if suffered to be retained long, likely to produce symptoms not *only* troublesome but very dangerous to the patient.

Since I communicated a case of Steatoma being cured by seton,* I have met with another of smaller size, which succeeded equally well, by the same treatment.

